# Matrine improves bile acid metabolism and reduces inflammatory and oxidative stress in colitis via the JAK2 pathway

**DOI:** 10.1186/s13020-026-01387-z

**Published:** 2026-03-26

**Authors:** Zhi-xian Jiang, Xue-liang Chen, Qi Sun, Li-chao Yang, Ya-wei Zhang, Qiang Wu, Heng-chang Yao, Dan Zhang, Lian-wen Yuan

**Affiliations:** 1 Department of Geriatric Surgery, The Second Xiangya Hospital, Central South University, 139 Renmin Middle Road, Changsha, Hunan People’s Republic of China; 2 Department of Anorectal Surgery, The First People’s Hospital of Chenzhou, Chenzhou, 423000 Hunan China; 3 Clinical Nursing Teaching and Research Section, The Second Xiangya Hospital, Central South University, Changsha, 410011 Hunan China

**Keywords:** Colitis, Matrine, Ulcerative colitis, Bile acid metabolism, JAK2

## Abstract

**Background:**

Dysbiosis during colitis alters the conversion of primary to secondary bile acids by gut microbiota, which affects bile acid receptor signaling and may exacerbate mucosal inflammation in experimental colitis models. While the natural compound matrine has known anti-inflammatory properties, its therapeutic mechanism in colitis remains unclear. This study aims to elucidate matrine’s potential by identifying its molecular targets and effects in colitis.

**Methods:**

Bioinformatics and molecular docking were used to identify potential drug targets. A multi-model approach was then employed, using a dextran sulfate sodium (DSS)-induced murine model of colitis, a lipopolysaccharide (LPS)-stimulated intestinal epithelial cell model, and clinical colon and serum samples from ulcerative colitis (UC) patients. The effects of matrine on inflammatory cytokines, oxidative stress markers, and bile acid levels were detected using ELISA and various commercial kits. Intracellular reactive oxygen species (ROS) were measured by flow cytometry.

**Results:**

JAK2 as a key hub target for matrine, and molecular docking predicted a direct binding interaction. The JAK2 level was found to be upregulated, while bile acid transporters and overall serum bile acid levels were decreased in human UC patients and correlated negatively with disease severity. In a DSS-induced murine model of colitis, matrine treatment mitigated disease symptoms, reduced key inflammatory markers (IL-1β, TNF-α, IL-6) and oxidative stress indicators (including ROS), and restored bile acid homeostasis by upregulating transporters (MRP3 and MRP4) and bile acid receptor FXR. Critically, the mechanism was confirmed to be JAK2-dependent in vitro; experiments demonstrated that JAK2 overexpression alone was sufficient to induce pathology and that it completely reversed the therapeutic effects of matrine.

**Conclusion:**

This study is the first to comprehensively demonstrate that matrine directly targets the JAK2/STAT3 signaling axis to restore bile acid homeostasis and suppress inflammation in experimental colitis with validation in human UC patients, providing novel mechanistic and translational insight into how matrine may benefit colitis.

## Introduction

Colitis refers to inflammation of the colon, the main part of the large intestine, and can be acute or chronic depending on the cause [1]. Colitis may result from a range of factors including infections, medications, ischemia, or immune-mediated processes, with symptoms such as abdominal pain, diarrhea, urgency, and bleeding [2]. In the context of chronic colitis, persistent inflammation disrupts mucosal integrity and bowel function, often requiring long-term medical management [3]. Ulcerative colitis is a subtype of chronic colitis characterized by continuous mucosal inflammation that begins in the rectum and extends proximally through the colon [4]. The pathogenesis of chronic colitis involves complex interactions among genetic susceptibility, immune dysregulation, environmental factors, and the gut microbiota [5]. Given that the etiology of colitis is multifactorial and that potential risk factors and pathophysiology vary from individual to individual, the development of effective, personalized therapeutic approaches is critical.

Bile acids are amphipathic molecules derived from liver cholesterol and microbiota-driven biotransformations in the colon [6]. Bile acid malabsorption has been well documented in certain colitis-related conditions (such as microscopic colitis) and as a contributor to diarrhea, and it is often under-recognized clinically [7]. Compared with healthy subjects, the bile acid pool size was significantly decreased in colitis patients, and this reduction was associated with disease activity [8]. Impaired bile acid metabolism also plays an important role in persistent diarrhea symptoms in colitis. Mechanisms include increased fecal water and electrolyte secretion, lipid indigestion, damage to epithelial tight junctions, and increased mucosal permeability [9]. Therefore, developing treatment for bile acid malabsorption is a promising strategy for colitis therapy.

As previously reported, NLRP3 inflammasome and IL-1β exert critical effects during colitis development, considering that the NLR family pyrin domain containing 3 (NLRP3) triggers intestinal inflammation in the mucosal layer, and increased IL-1β level is associated with non-responsiveness to anti-TNF therapy [10]. Matrine is a biologically active quinolizidine alkaloid isolated from traditional Chinese herbs, including *Sophoraalopecuroides*, *Sophoraflavescens*, and *Sophoratokinensis* [11]. Matrine has been revealed to have numerous biological functions, including antifibrosis, antitumor, and antiproliferation [12–14]. Furthermore, matrine has anti-inflammatory and antioxidative functions [15]. Moreover, matrine has been reported to suppress NLRP3 inflammasome signaling in different diseases, including porcine reproductive and respiratory syndrome virus (PRRSV) [16], sepsis [17] and advanced glycation end products (AGEs)-induced cardiac injury [18]. These findings suggest that matrine might also inhibit pyroptosis to alleviate colitis, with its role in lipid profile changes yet to be determined.

In this study, drug target and disease target genes were analyzed and compared, and the overlapping target gene JAK2 was identified. The dextran sulfate sodium (DSS)-induced murine colitis model was established, and the in vivo effects of matrine on intestinal pathology, inflammatory function, JAK2 expression, cholesterol levels, and bile acid metabolism in experimental colitis mice were investigated. In vitro, mouse intestinal epithelial cells MODE-K were stimulated with LPS and treated with matrine, and examined for intestinal epithelial cell functions, inflammatory factors, bile acid metabolism-associated proteins, and JAK2 signaling pathway alterations. Collectively, this study provides robust bioinformatics analyses and an experimental basis for understanding the effects of matrine on bile acid metabolism, inflammatory responses, and JAK2 alterations, while improving intestinal epithelial cell function and colitis-like features.

## Materials and methods

### Experimental animals

A total of 36 female C57BL/6 mice (6–8 week-old) were procured from the SLAC experimental animal center (Changsha, China), and were kept in a controlled environment with consistent conditions, including a humidity level of 50–60%, a temperature of 22–24 °C, and a light–dark cycle of 12 h for one week during which all mice were given ad libitum access to normal chow diet and water. All procedures were carried out under the approval of the Animal Care and Use Committee of the Second Xiangya Hospital of Central South University.

### Dextran sulfate sodium (DSS)-induced murine model of colitis

Mice were randomly assigned into six groups (n = 6 in each group): PBS, DSS, and DSS + Matrine-L, DSS + Matrine-M, DSS + Matrine-H, DSS + 5-ASA. The IBD model was established as previously described [19]. Each mouse in the DSS, DSS + matrine, and DSS + 5-ASA groups was given a 7-day treatment with 3% DSS in the drinking water, and the normal control mice received distilled water ad libitum. The DSS + Matrine-L, DSS + Matrine-M, and DSS + Matrine-H mice received 50, 100, 200 mg/kg/day matrine (Yuanye Biotech, Shanghai, China) by oral gavage [20], DSS + 5-ASA mice received 5-ASA (Sigma-Aldrich, St. Louis, MO) 100 mg/kg/day by oral gavage. The survival and body weight of mice were observed and recorded once daily. Colon and blood samples were harvested after anesthetizing and sacrificing mice on day 8, and the harvested tissue was used for immunoblotting and histological analysis. The blood samples were used for serum biochemistry analysis. For histological analysis, colon tissue samples were fixed in 4% paraformaldehyde, paraffin-embedded, sectioned at 5 mm, mounted on glass slides, and deparaffinized. Slices were stained with the hematoxylin and eosin (H&E) kit (Solarbio, Beijing, China), and a light microscope was used to observe them.

### Disease activity index (DAI) scoring

Colitis severity was assessed by measuring body weight, stool consistency, and blood in the stool, as per the previously established grading system [21]. In short, scores were assigned for weight loss (0: 0–1%, 1: 1–5%, 2: 5–10%, 3: 10–20%, 4: > 20%), stool consistency (0: Normal, 2: Loose stool, 4: Diarrhea), and presence of blood within the stool (0: Negative, 2: Visual blood in stool, 4: Fresh rectal bleeding).

### Immunohistochemical staining (IHC staining)

After rehydrating tissue slices, 3% hydrogen peroxide was applied to quench endogenous peroxidases, followed by an overnight incubation at 4 °C using primary antibodies against JAK2 (bs-0908R, Bioss, Woburn, USA) or a rabbit IgG (isotype control, ab37415, Abcam, Cambridge, UK). The slices were washed in PBS, then incubated for 30 min at 37 °C with the poly-IgG-HRP antibody (Boster, Wuhan, China). After 10 min of staining with a diaminobenzidine (DAB) kit (Boster), slices were visualized under a microscope (Olympus, Tokyo, Japan). The histological results were reviewed by two independent, blinded pathologists.

### Immunoblotting

A BCA Protein Assay Kit (Beyotime, Shanghai, China) was used to determine protein levels in specimens collected from cells and tissues. Following electrophoresis on 10–14% SDS-PAGE, the separated proteins were electroblotted onto a PVDF membrane. Membrane was blocked within Odyssey blocking buffer (LI-COR Bioscience, Lincoln, USA) for 1 h at room temperature (RT), and then incubated with primary antibodies against p-JAK2 (AP0917, Abclonal, Woburn, USA), JAK2 (AF6022, Affinitiy Bioscience, Changzhou, China), MRP3 (bs-0656R, Bioss, Beijing, China), MRP4 (DF6921, Affinity Bioscience), IL-1β [12242, Cell Signaling Technology (CST), Danvers, USA], TNF-α (11948, CST), IL-6 (CSB-PA06757A0RB, CUSABIO, Wuhan, China), p-STAT3 (AF3293, Affinity Bioscience), STAT3 (10253-2-AP, Proteintech, Wuhan, China), p-STAT1 (28977-1-AP, Protientech), STAT1 (10144-2-AP, Proteintech), FXR (M022312, Abmart, Shanghai, China), and GAPDH (endogenous control, 60004-1-Ig, Proteintech) overnight at 4 °C (dilution 1:1000). A horseradish peroxidase-labeled goat anti-rabbit immunoglobulin G (IgG) (sc-1004; Santa Cruz Biotechnology, Santa Cruz, USA), or horse anti-mouse IgG (7076S; CST) (dilution 1:2000) was used to detect primary antibody, and the Enhanced Chemiluminescence (ECL) detection system (Promega, Madison, USA) was used to visualize results.

### Total cholesterol and bile acid determination

For serum total cholesterol (T-CHO) and bile acid determination, the T-CHO assay kit and bile acid assay kit (A111-1-1 and E003-2-1, Nanjing Jiancheng Biotech, Nanjing, China) were used. After settling for 2 h, the blood was centrifuged for 30 min at 13,523 × g at 4 °C. The collected supernatant serum was used according to the protocols outlined in the kit manuals. The T-CHO and Bile acid serum levels were measured at 500 nm for T-CHO and 405 nm for bile acids, and the results were compared to the respective standard curves.

### ELISA

About 100 mg of mouse colon tissues were homogenized in 1 ml of cooled PBS using a tissue homogenizer (Thermo Fisher Scientific, Waltham, USA). The homogenates were subsequently centrifuged for 5 min at 5000 g, 4 ℃. The supernatant was collected and subjected to ELISA with different commercial mouse ELISA kits: IL-1β (CSB-E08054m, CUSABIO), TNF-α (CSB-E04741m, CUSABIO), and IL-6 (CSB-E04639m, CUSABIO). After adding the stop solution to each well, the OD at 450 nm was measured with a Shimadzu Corporation UV spectrophotometer (Tokyo, Japan). The final concentrations of IL-1β, TNF-α, and IL-6 in the colon tissues were calculated using the respective standard curves.

### Myeloperoxidase (MPO), nitric oxide (NO), and malondialdehyde (MDA) measurement

To assess levels of MPO, NO, and MDA in colonic tissues, standardized procedures were followed using kits from Nanjing Jiancheng Biotech (A044-1-1, A012-1-2, A003-1-2). For MPO activity, 50 mg of colon tissue was rinsed, homogenized in cooled PBS (w/w 1:19), and the total protein concentration was determined using a BCA assay kit. The MPO activity was then measured according to the kit's protocols at 460 nm using a UV spectrophotometer. Similarly, for NO content, 50 mg of colon tissue was processed in the same manner, and NO levels were measured by quantifying the stable metabolites nitrite (NO2 −) and nitrate (NO3 −) at 550 nm. Lastly, for MDA levels, 100 mg of colon tissue was used, following a similar procedure of washing and homogenization in cooled PBS, with MDA levels measured at 532 nm.

### Reactive oxygen species (ROS) detection

Intracellular ROS levels were detected using a Reactive Oxygen Species Assay Kit with a DCFH-DA probe (S0033S, Beyotime). After experimental treatments, MODE-K cells were collected and incubated with 10 µM DCFH-DA diluted in serum-free medium for 20 min at 37 °C in light-deprived conditions. After incubation, the cells were washed three times with PBS. The fluorescence intensity was immediately measured using a flow cytometer (Novocyte, Agilent Technologies, Santa Clara, USA).

### Cell line and cell cultivation

Mouse intestinal epithelial cell line, MODE-K, was procured from Shanghai GuanDao Biological Engineering (Shanghai, China) and cultivated within Eagle’s Minimum Essential Medium containing 10% FBS (Invitrogen, Carlsbad, USA), 2 mM L-glutamine, 100 IU penicillin, and 100 mg/ml streptomycin at 37 °C in a humidified incubator with 5% CO_2_. For LPS treatment, MODE-K cells were stimulated with 200 ng/mL LPS for 48 h. For matrine treatment, MODE-K cells were subjected to 24- or 48-h treatment using 1, 2, and 3 mg/ml.

### Cell transfection and treatment

For JAK2 overexpression, OriTrans-LV (Oribio, Changsha, China) was used to facilitate transfection of lentivirus encoding a mouse JAK2-coding sequence fragment (JAK2 oe) into MODE-K cells. The cells were collected for subsequent analysis after 48 h. An empty lentivirus was utilized as a negative control.

### Clinical samples and ethics statement

A total of 12 patients with ulcerative colitis (UC) and 12 healthy control subjects were recruited from the Second Xiangya Hospital of Central South University. The diagnosis of UC was confirmed based on clinical, endoscopic, and histopathological findings. For UC patients, disease activity was assessed using the Mayo score as previously described [22]. Colon biopsy tissues and corresponding peripheral blood serum samples were collected from all participants. This study was approved by the Ethics Committee of the Second Xiangya Hospital of Central South University (Approval No.: 2017-S117), and written informed consent was obtained from all subjects prior to sample collection.

### Bioinformatics analysis

SwissTargetPrediction (http://www.swisstargetprediction.ch/) was used to predict matrine-targeted genes. The chemical structure of matrine was downloaded from PubChem (PubChem ID: 91466, https://pubchem.ncbi.nlm.nih.gov/). The inflammatory bowel disease (IBD)-associated genes were selected from GeneCard (https://www.genecards.org/). The matrine targeted IBD-associated genes were subjected to enrichment analysis by the online tool Metascape (https://metascape.org). The GSE179285 (contained expression profile of 47 Crohn’s disease (CD) inflamed intestinal tissues and 31 normal intestinal tissues) and GSE67106 (contained 21 CD intestinal tissues and 22 normal intestinal tissues) were downloaded from the GSE database (https://www.ncbi.nlm.nih.gov/geo/). The different expression genes were selected by∣logFC∣ > 0.6, p-value < 0.05.

### Molecular docking

The 3D crystal structure of matrine was obtained from the PubChem database and converted to PDB format using Chem3D software. The JAK2 protein structure (PDB ID: 2W1I) was retrieved from the UniProt database (https://www.uniprot.org/). Molecular docking between matrine and JAK2 was performed using AutoDock4, and the docking poses were visualized in PyMOL (version 3.1).

### Statistical analysis

GraphPad software was applied to analyze data. All data were represented in terms of the means ± standard deviation. One-way ANOVA followed by Tukey’s post hoc test was applied to analyze three or more groups. Student’s* t*-test was applied to contrast the differences between the two experimental groups. A *P*-value of less than 0.05 was deemed statistically significant.

## Results

### Matrine (drug)/disease target gene analyses

**Fig. 1:**
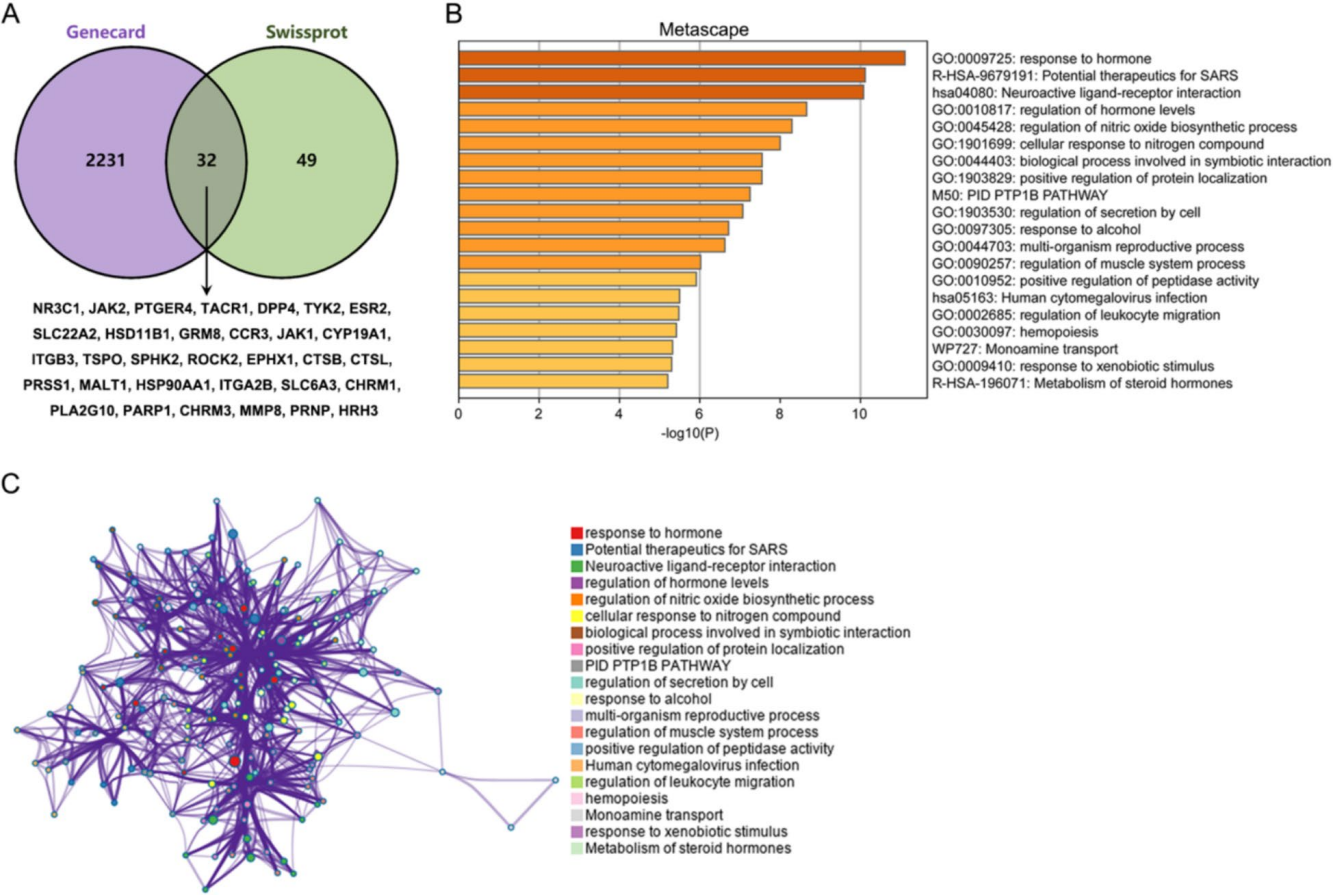
Matrine (drug)/disease target gene analyses **A** matrine (PubChem ID: 91466) target gene prediction was performed in SwissTargetPrediction (http://www.swisstargetprediction.ch/). Disease target genes were obtained by searching “inflammatory bowel diseases (IBD)” in Genecard. Genes obtained were compared and 32 overlapping drug/disease targets were obtained. **B**, **C** Gene Ontology (GO) enrichment annotation was performed using Metascape

For identifying drug/disease target genes, the SwissTargetPrediction (http://www.swisstargetprediction.ch/) website was used, and searching for “inflammatory bowel diseases (IBD)” was performed on Genecard. “matrine” (PubChem ID: 91466) retrieved 81 target genes (probability > 0) from SwissTargetPrediction. A search for IBD as a keyword from Genecard yielded 2263 associated genes; comparison with SwissTargetPrediction predicted matrine binding genes yielded 32 overlapping target genes (NR3C1, JAK2, PTGER4, TACR1, DPP4, TYK2, ESR2, SLC22A2, HSD11B1, GRM8, CCR3, JAK1, CYP19A1, ITGB3, TSPO, SPHK2, ROCK2, EPHX1, CTSB, CTSL, PRSS1, MALT1, HSP90AA1, ITGA2B, SLC6A3, CHRM1, PLA2G10, PARP1, CHRM3, MMP8, PRNP, HRH3) (Fig. 1A). Gene Ontology (GO) enrichment annotation and KEGG was performed using Metascape. Figure 1B, C shows that these genes were significantly enriched upon hormone/modulation of hormone levels, immune cell migration, and steroid metabolism, which was consistent with the complex alterations of bile acid and cholesterol during IBD.

**Fig. 2:**
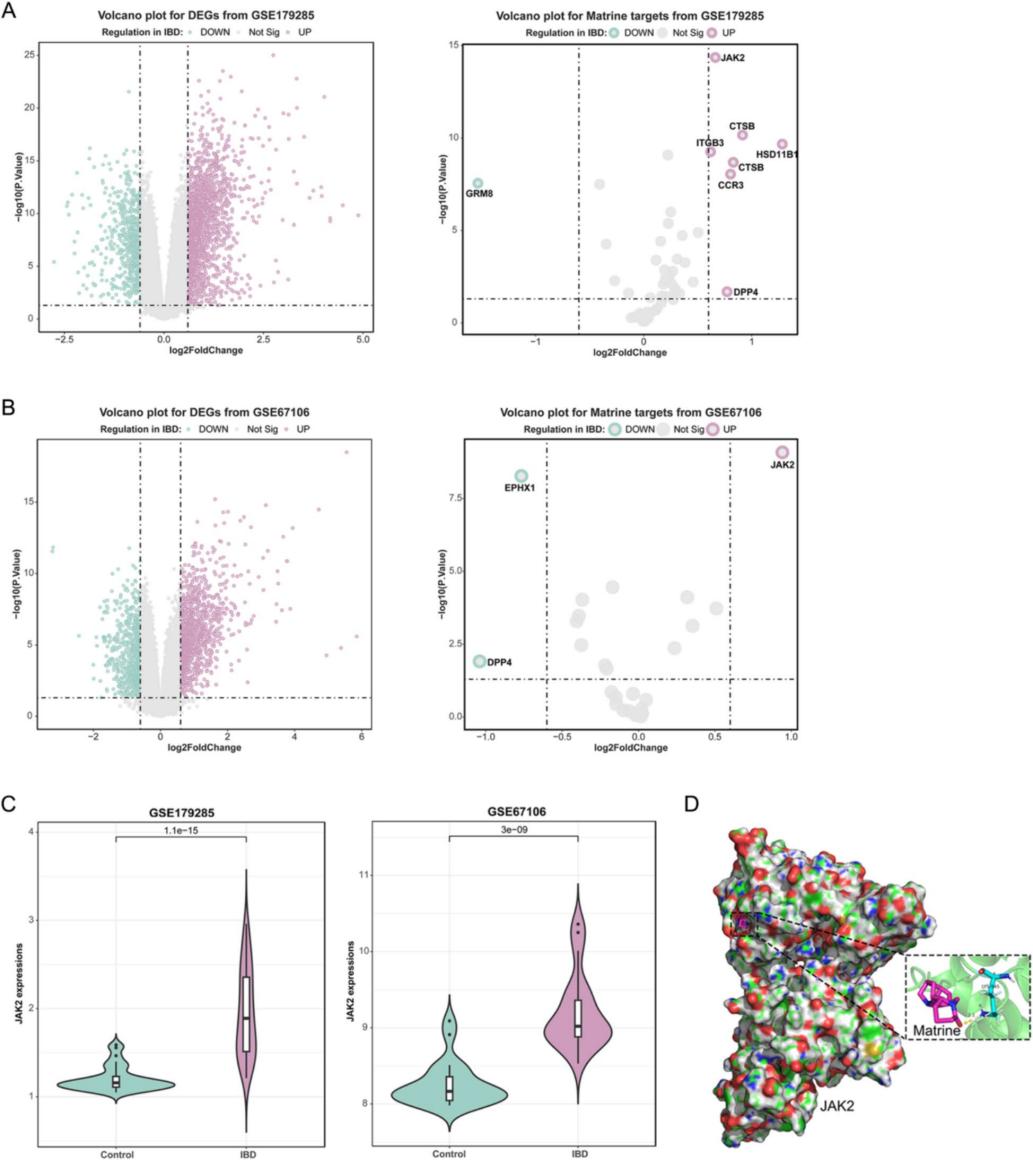
Differentially expressed genes (DEGs) in IBD **A** DEGs in 47 inflammatory Crohn’s disease (CD) intestinal tissues and 31 control intesintal tissues were compared according to GSE179285. **B** DEGs in 21 patients with intestinal tissues from inflammatory CD and 22 control intestinal tissues were compared using GSE67106. **C** JAK2 expression in IBD and control samples according to GSE179285 and GSE67106. **D** Molecular docking simulation showing the binding conformation of matrine and the JAK2 protein (PDB ID: 2W1I). The binding interaction energy between matrine and JAK2 was –5.49 kcal/mol

DEGs in IBD were subsequently analyzed using two datasets, GSE179285 and GSE67106. Differential expression analysis based on GSE179285 yielded 761 down-regulated (logFC < − 0.6, p value < 0.05) and 1313 up-regulated genes (logFC > 0.6, p value < 0.05). Of these DEGs, 8 were among the 32 target genes obtained above, with 7 up-regulated, including JAK2 (logFC = 0.663, p value = 4.39e-15), and 1 down-regulated, GRM8 (logFC = − 1.533, p value = 2.78e-08) (Fig. 2A). GSE67106 yielded 619 down-regulated (logFC < − 0.6, p value < 0.05) and 1908 up-regulated genes (logFC > 0.6, p value < 0.05). Of them, 3 were among the 32 target genes, including JAK2 (logFC = 0.942, p value = 8.21e-10) (Fig. 2B). The expression level of JAK2 showed to be dramatically increased within IBD than normal controls according to GSE179285 and GSE67106 (Fig. 2C). Furthermore, molecular docking analysis predicted a stable binding between matrine and JAK2 (Fig. 2D). The binding interaction energy between matrine and JAK2 was –5.49 kcal/mol, with contributions from van der Waals interaction energy (–4.69 kcal/mol) and electrostatic interaction energy (–0.80 kcal/mol).

### Matrine alleviated the pathological changes in experimental colitis mice and reduced JAK2 expression

**Fig. 3:**
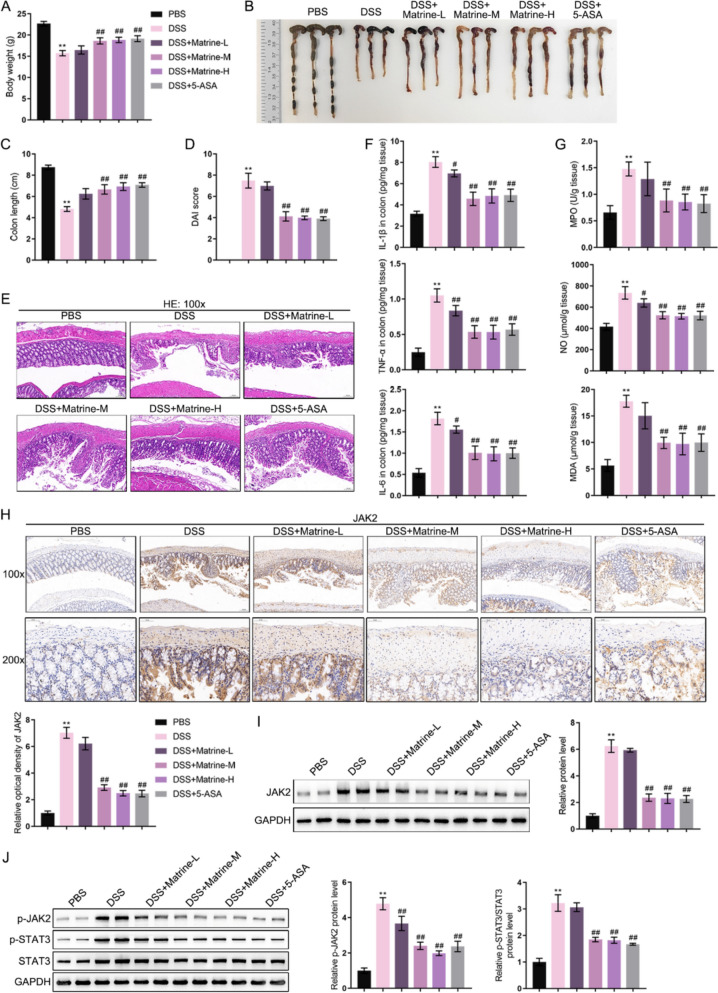
Effects of matrine on intestinal pathology, inflammatory function, and JAK2 expression in experimental colitis mice Dextran Sulfate Sodium (DSS)-induced murine model of colitis was established in mice; matrine and 5-ASA treatment was administered as described. **A**–**C** Body weight and colon length were measured. **D**, **E** Histopathological alterations in colon tissues were evaluated using **H**&**E** staining; DAI scores were assigned according to the staining. **F**, **G** The levels of IL-1β, TNF-α, IL-6, MPO, NO, and MDA in colon tissues were examined using commercial assay kits. **H**, **I** The protein levels of JAK2 in mice colon tissues were determined using Immunohistochemical staining (IHC staining) and Immunoblotting. Magnification = 100 × or 200 × for IHC staining. **J** The protein levels of p-JAK2, p-STAT3, and STAT3 were detected using Immunoblotting. **p < 0.01, vs. PBS group; #p < 0.05, ##p < 0.01 vs. DSS group

To investigate the specific functions of matrine and its underlying mechanism, a DSS-induced murine colitis model was established, and matrine was administered as described. Compared to normal controls, the body weight and colon length of experimental colitis mice were dramatically reduced; as compared to the DSS mice, matrine in medium, high concentration partially reversed the model mice's body weight and colon length, as 5-ASA (a therapeutic drug commonly used for colitis) did (Fig. 3A–C). Consistently, experimental colitis mice saw a significantly high DAI score, which was significantly decreased by Matrine-M, Matrine-H, or 5-ASA treatment (Fig. 3D). Regarding histopathological alterations in colon tissues, H&E staining revealed that the intestinal structure of DSS mice was disorganized and incomplete with inflammation, which was partially ameliorated by Matrine-M, Matrine-H or 5-ASA treatment (Fig. 3E). Regarding inflammation, the IL-1β, TNF-α, and IL-6 levels showed to be dramatically increased within model mice colon tissues than normal controls, whereas partially decreased within the Matrine-M, Matrine-H, or 5-ASA treatment mice than the DSS mice (Fig. 3F). Regarding oxidative stress, model mice colon tissues also saw remarkably elevated MPO, NO, and MDA levels than normal controls, which were partially decreased by matrine treatment than the DSS mice (Fig. 3G). Moreover, JAK2 protein levels were dramatically increased in model mouse colon tissues compared to normal controls, and were significantly reduced by Matrine-M, Matrine-H, or 5-ASA treatment compared to DSS mice (Fig. 3H, I). Regarding the JAK2/STAT3 signaling, compared to normal mice, JAK2 and STAT3 phosphorylation levels were increased in DSS mice; whereas Matrine-M, Matrine-H, or 5-ASA treatment partially decreased JAK2 and STAT3 phosphorylation levels (Fig. 3J).

### Matrine modulated bile acid metabolism in experimental colitis mice

**Fig. 4:**
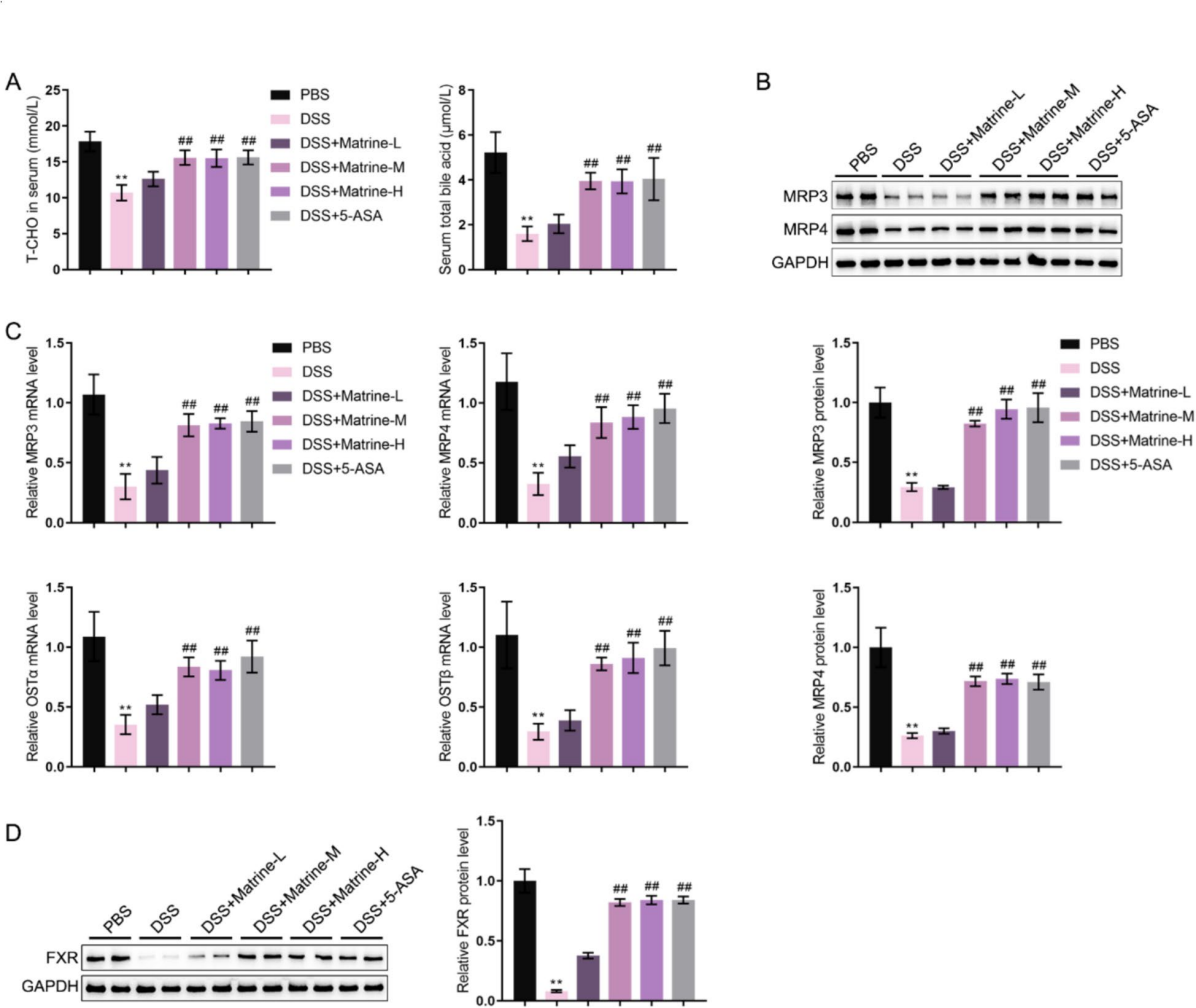
Effects of matrine on cholesterol metabolism in experimental colitis mice **A** Serum levels of total cholesterol (T-CHO) and bile acid were examined using commercial assay kits. **B** The protein levels of MRP3 and MRP4 in colon tissues were examined using Immunoblotting. **C** The mRNA levels of MRP3, MRP4, OSTα and OSTβ in colon tissues were examined using qRT-PCR. **D** The protein levels of FXR in colon tissues were examined using Immunoblotting. **p < 0.01, vs. PBS group; ##p < 0.01 vs. DSS group

Regarding the cholesterol metabolism in experimental colitis mice, DSS mice saw significantly lower total cholesterol (T-CHO) and total bile acid serum levels, which were partially increased by Matrine-M, Matrine-H, or 5-ASA treatment compared with the DSS group (Fig. 4A). The protein levels of bile acid metabolism-related factors, MRP3, MRP4, OSTα, and OSTβ in colon tissues were monitored. DSS stimulation dramatically reduced the protein levels of MRP3 and MRP4, and the mRNA levels of MRP3, MRP4, OSTα, and OSTβ in model mouse colon tissues. In contrast, Matrine-M, Matrine-H, or 5-ASA treatment partially increased MRP3, MRP4, OSTα, and OSTβ levels compared with the DSS group (Fig. 4B, C). Moreover, FXR is a bile acid-activated nuclear receptor that plays a pivotal role in regulating bile acid metabolism, intestinal homeostasis, and barrier function. Its activation promotes expression of target genes involved in bile acid transport and anti-inflammatory signaling, whereas its reduced activity is associated with increased intestinal inflammation in IBD models [23, 24]. Hence, the protein levels of FXR in colon tissues were detected. Compared with normal mice, the FXR protein level was decreased in DSS mice; whereas Matrine-M, Matrine-H, or 5-ASA treatment partially increased the FXR protein level (Fig. 4D).

### JAK2 overexpression exacerbates LPS-induced inflammation, oxidative stress, and bile acid metabolism dysfunction in intestinal epithelial cells

**Fig. 5:**
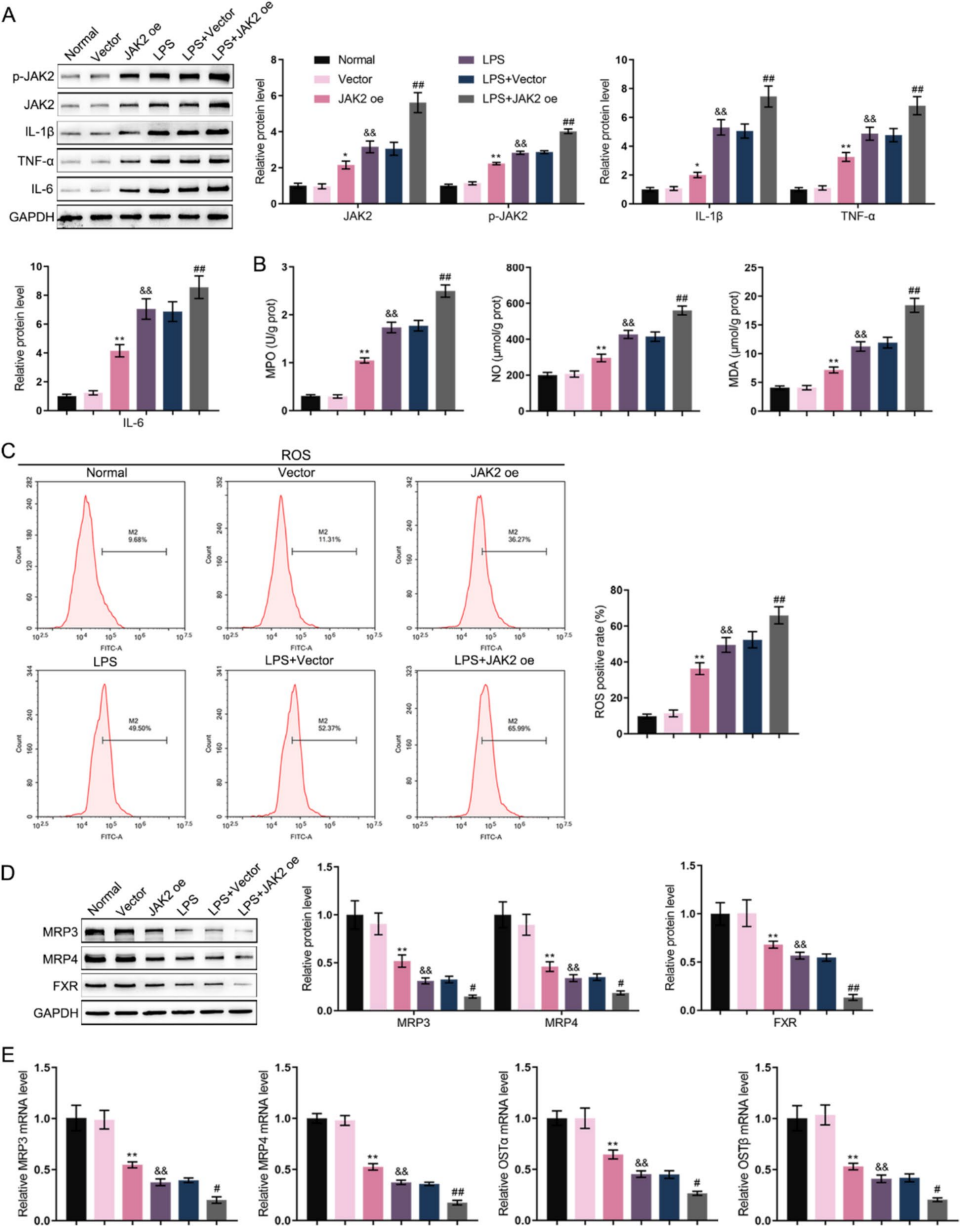
Effects of JAK2 overexpression on intestinal epithelial cells upon inflammation MODE-K cells were transfected with a JAK2-overexpressing plasmid (JAK2 oe) or a control vector, with or without subsequent LPS stimulation. **A** The protein levels of JAK2 and p-JAK2 and the inflammatory factors IL-1β, TNF-α, and IL-6 were determined by Immunoblotting. **B** Cellular levels of MPO, NO, and MDA were measured using biochemical kits. **C** Intracellular ROS production was assessed by DCFH-DA staining and quantified with flow cytometry. **D** The protein levels of the bile acid metabolism-related factors FXR, MRP3 and MRP4 were examined by Immunoblotting. **E** The mRNA levels of MRP3, MRP4, OSTα, and OSTβ were measured by qRT-PCR. **p < 0.01 vs. Vector group; ##p < 0.01 vs. Vector group; &&p < 0.01 vs. LPS + Vector group

To further confirm the direct role of JAK2 in intestinal epithelial cells, JAK2 was overexpressed in MODE-K cells, and its effects were observed with and without LPS stimulation. The results showed that JAK2 overexpression alone was sufficient to significantly increase the protein levels of JAK2 and JAK2 phosphorylation, as well as the pro-inflammatory cytokines IL-1β, TNF-α, and IL-6, which were significantly increased in cells that were also treated with LPS (Fig. 5A). Similarly, JAK2 overexpression led to a notable increase in the levels of the oxidative stress markers MPO, NO, and MDA (Fig. 5B) and intracellular ROS (Fig. 5C). This effect was further exacerbated by LPS stimulation. Conversely, JAK2 overexpression significantly downregulated the protein levels of MRP3, MRP4, and FXR (Fig. 5D), as well as the mRNA levels of MRP3, MRP4, OSTα, and OSTβ (Fig. 5E). This suppression of bile acid metabolism-related factors was more pronounced in cells co-treated with LPS (Fig. 5D, E). These findings demonstrate that JAK2 directly promotes inflammation and oxidative stress while disrupting bile acid transporter expression in intestinal epithelial cells.

### Matrine inhibited LPS-induced inflammation in intestinal epithelial cells

**Fig. 6:**
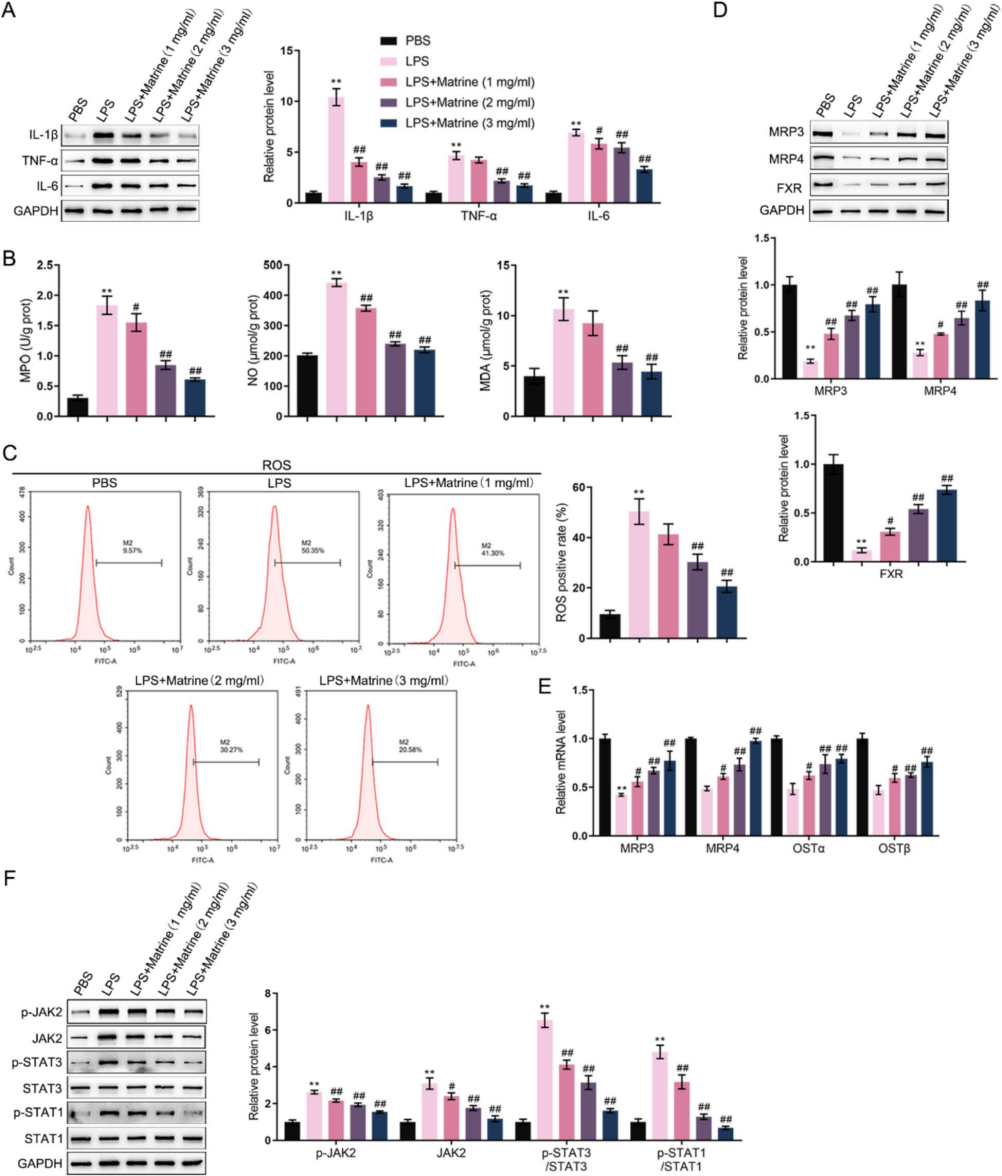
Effects of matrine on intestinal epithelial cell function upon inflammation Mouse intestinal epithelial cell line, MODE-K, was stimulated with LPS with or without matrine treatment (1, 2, 3 mg/ml), and examined for the protein levels of IL-1β, TNF-α, and IL-6 using Immunoblotting (**A**); The levels of MPO, NO, and MDA using commercial kits (**B**); Intracellular ROS levels were detected by flow cytometry (**C**); The protein levels of FXR, MRP3 and MRP4 were examined using Immunoblotting (**D**); The mRNA levels of MRP3, MRP4, OSTα and OSTβ were examined using qRT-PCR (**E**); The protein levels of p-JAK2, JAK2, p-STAT3, STAT3, p-STAT1, and STAT1 using Immunoblotting (**F**). **p < 0.01, vs. PBS group; #p < 0.05, ##p < 0.01 vs. LPS group

After confirming matrine improvement of IBD-like features in model mice, the in vitro effects of matrine on a mouse intestinal epithelial cell line, MODE-K, were investigated. MODE-K cell line was subjected to LPS stimulation in the presence or absence of matrine treatment (1, 2, 3 mg/ml) and examined for cell phenotype alterations. Regarding inflammation, LPS dramatically elevated IL-1β, TNF-α, and IL-6 protein contents, whereas matrine treatment decreased these pro-inflammatory cytokines dose-dependently (Fig. 6A). Regarding oxidative stress, LPS stimulation significantly increased the levels of MPO, NO, and MDA (Fig. 6B), as well as intracellular ROS; these effects were remarkably decreased by matrine treatment in a dose-dependent manner (Fig. 6C). Regarding bile acid metabolism, MRP3, MRP4, OSTα, and OSTβ mRNA or protein levels were determined. MRP3, MRP4, and FXR protein levels in intestinal epithelial cells were dramatically decreased by LPS stimulation, whereas increased by matrine treatment dose-dependently (Fig. 6D). Moreover, the mRNA levels of MRP3, MRP4, OSTα, and OSTβ were decreased by LPS and increased by matrine treatment (Fig. 6E). Regarding the JAK2/STAT3 signaling, LPS stimulation significantly elevated JAK2 protein contents. They enhanced the phosphorylation of JAK2, STAT3, and STAT1. In contrast, matrine treatment partially decreased JAK2 levels and suppressed JAK2, STAT3 and STAT1 phosphorylation (Fig. 6F).

### Dynamic effects of matrine and JAK2 on intestinal epithelial cell function upon inflammation

**Fig. 7:**
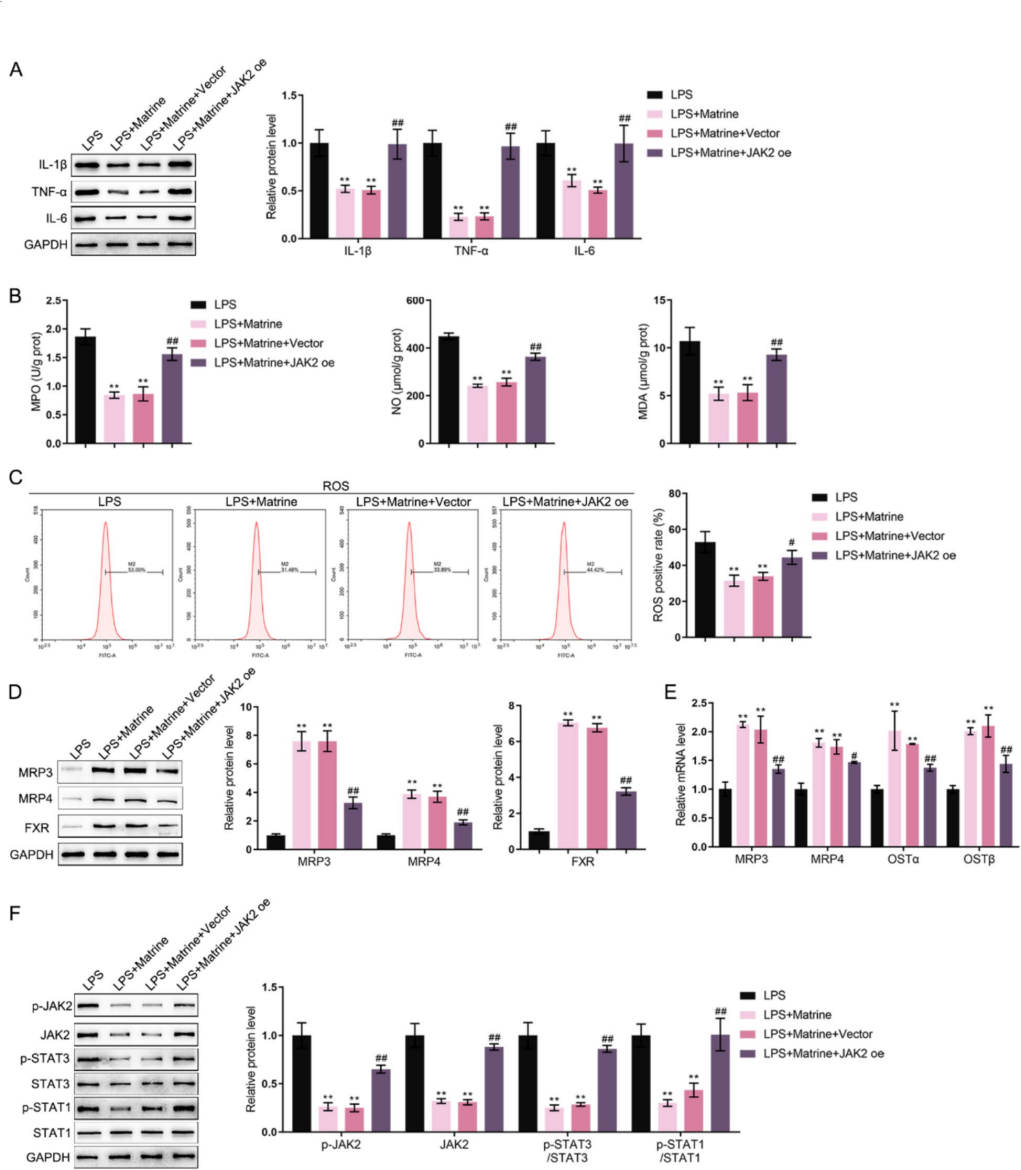
Dynamic effects of matrine and JAK2 on intestinal epithelial cell function upon inflammation MODE-K cells were stimulated with LPS with or without matrine treatment (2 mg/ml), transfected with JAK2-overexpressing plasmid (JAK2 oe), and examined for the protein levels of IL-1β, TNF-α, and IL-6 using Immunoblotting (**A**); the levels of MPO, NO, and MDA using commercial kits (**B**); Intracellular ROS levels were detected by flow cytometry (**C**); The protein levels of FXR, MRP3 and MRP4 were examined using Immunoblotting (**D**); The mRNA levels of MRP3, MRP4, OSTα and OSTβ were examined using qRT-PCR (**E**); the protein levels of JAK2, p-STAT3, STAT3, p-STAT1, and STAT1 using Immunoblotting (**F**). **p < 0.01, vs. LPS group; #p < 0.05, ## p < 0.01 vs. LPS + matrine + vector group

Since JAK2 has been recognized as a hub gene in matrine’s role in IBD, the dynamic effects of matrine and JAK2 were finally explored in the LPS-stimulated MODE-K cell line with or without matrine treatment (2 mg/ml). Regarding the protein contents of pro-inflammatory cytokines, JAK2 overexpression partially abolished the suppressive effects of matrine upon the levels of IL-1β, TNF-α, and IL-6 (Fig. 7A). Regarding oxidative stress, JAK2 overexpression also partially eliminated the suppressive effects of matrine upon MPO, NO, and MDA levels (Fig. 7B), as well as on intracellular ROS (Fig. 7C). As for bile acid metabolism, matrine increased the protein levels of MRP3, MRP4 and FXR along with the mRNA levels of MRP3, MRP4, OSTα, and OSTβ, whereas JAK2 overexpression significantly decreased the levels of these factors (Fig. 7D, E). Regarding JAK2 signaling, JAK2 overexpression significantly attenuated the suppressive effects of matrine on JAK2 protein levels and JAK2/STAT3/STAT1 phosphorylation (Fig. 7F).

### JAK2 is upregulated, and bile acid metabolism is dysregulated in clinical UC samples

**Fig. 8:**
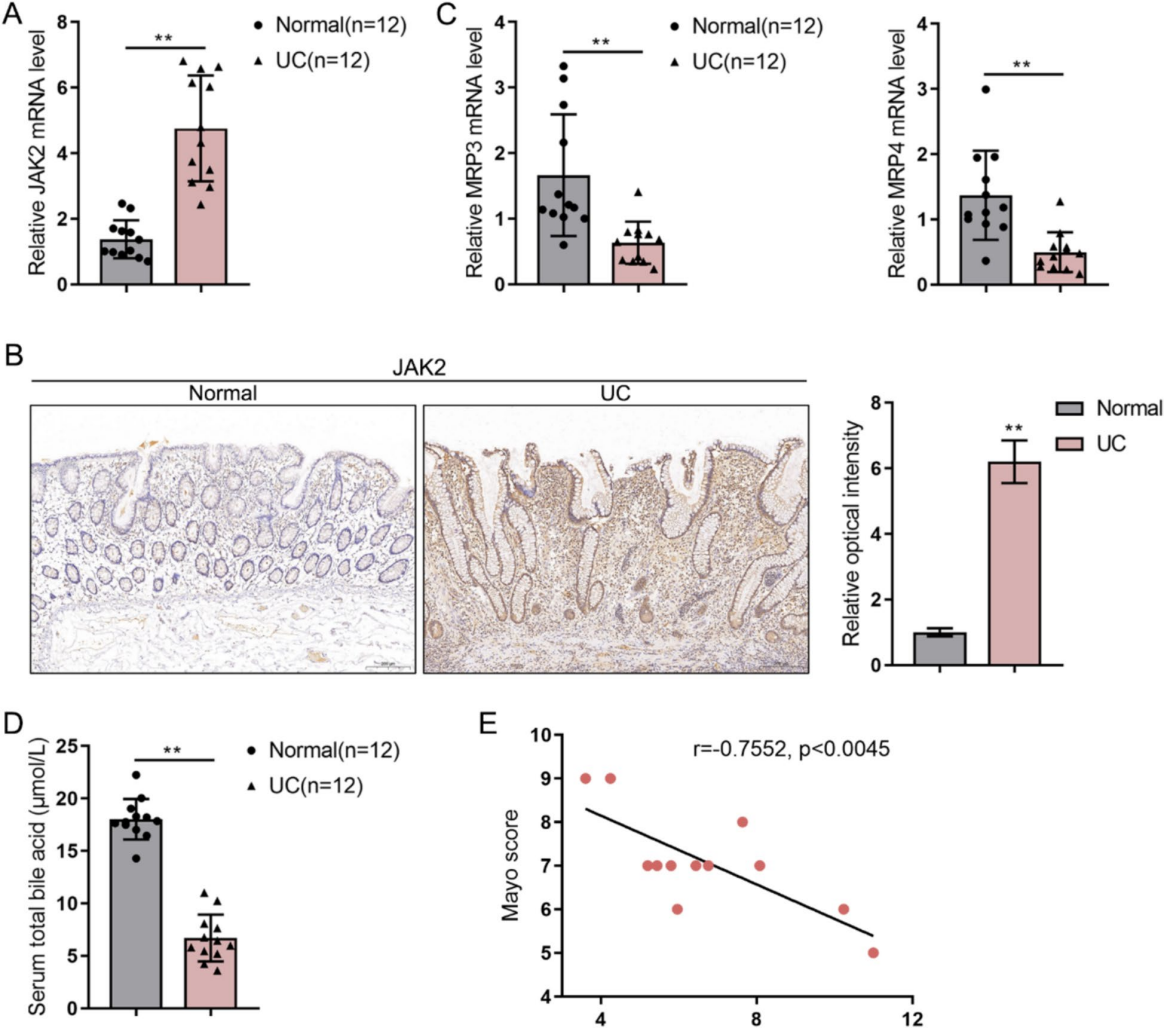
Analysis of JAK2 expression and bile acid metabolism in clinical UC samples Colon biopsy tissues and serum were collected from healthy controls (n = 12) and patients with UC (n = 12). **A** Relative mRNA expression of JAK2 in colon tissues was measured by qRT-PCR. **B** Representative IHC images and quantification showing JAK2 protein levels in colon tissues. **C** Relative mRNA expression of bile acid transporters MRP3 and MRP4 in colon tissues was measured by qRT-PCR. **D** Serum total bile acid levels were measured. **E** The correlation between serum total bile acid levels and Mayo scores in UC patients. **p < 0.01 vs. Normal group

To validate the findings from preclinical models, colon biopsies and serum were collected from UC patients and healthy controls. The analysis of these clinical samples revealed that the mRNA expression of JAK2 was significantly elevated in the colon tissues of UC patients compared to healthy individuals (Fig. 8A). At the protein level, IHC analysis showed a significantly higher abundance of JAK2 in UC tissues (Fig. 8B). Conversely, the mRNA levels of the bile acid transporters MRP3 and MRP4 were significantly lower in the colon tissues of UC patients (Fig. 8C). Consistent with this dysregulation of bile acid transport, the total bile acid levels in the serum of UC patients were significantly reduced compared to healthy controls (Fig. 8D). Importantly, lower serum bile acid levels were found to be negatively correlated with the Mayo score, which is a clinical measure of disease severity (Fig. 8E). These clinical data support the experimental findings, suggesting a link between JAK2 upregulation and impaired bile acid metabolism in the pathophysiology of human UC.

## Discussion

Herein, for the first time, the therapeutic potential and novel mechanism of matrine in colitis were explored through comprehensive gene-target and effect analyses. Using SwissTargetPrediction and Genecard, 32 overlapping target genes were identified between matrine and IBD, with molecular docking analysis further predicting a direct binding interaction between matrine and the hub gene JAK2. In the DSS-induced murine model of colitis, matrine improved body weight, colon length, and histopathological conditions while reducing inflammatory markers (IL-1β, TNF-α, IL-6) and oxidative stress indicators (MPO, NO, MDA). Matrine also modulated bile acid metabolism, evidenced by altered serum bile acid levels and the expression of transport-related factors MRP3, MRP4, OSTα, and OSTβ, and bile acid receptor FXR. These beneficial effects extended to MODE-K intestinal epithelial cells under LPS stimulation, where matrine reduced inflammation, oxidative stress (including intracellular ROS), improved bile acid homeostasis, and inhibited JAK2 signaling. Mechanistically, JAK2 overexpression alone sufficed to induce an inflammatory state and disrupt bile acid transport. Correspondingly, the therapeutic effects of matrine on LPS-treated cells were significantly attenuated by JAK2 overexpression, highlighting the crucial role of JAK2 in matrine's action against colitis. Crucially, these bioinformatics findings were validated in a clinical context, where samples from UC patients showed significantly upregulated JAK2 expression, decreased serum total bile acid levels, and downregulated expression of bile acid transporters MRP3 and MRP4 and the bile acid receptor FXR; notably, bile acid levels were negatively correlated with disease severity.

In our investigation of matrine’s therapeutic potential in colitis, bioinformatics identified JAK2 as a key hub gene among 32 overlapping drug-disease targets. This link was strengthened by molecular docking simulations, which predicted a direct and stable binding interaction between the matrine and JAK2 proteins. The clinical relevance of targeting this pathway was powerfully validated in human UC patients, where JAK2 was confirmed to be significantly upregulated in colon tissues. This is particularly significant because JAK2/STAT3 signaling exerts crucial effects on the adaptive and innate immune responses of the gut mucosa, contributing to colitis pathogenesis [25, 26]. Reportedly, this pathway could exert a critical effect on maintaining the balance between protective regulatory T cells and pathogenic effector T cells, as well as myeloid modulation, and intestinal epithelial cells to ameliorate the effective immunity [27]. Therefore, matrine's effects might be mediated through these key regulators. Our clinical data further revealed a novel connection where this upregulation of JAK2 is accompanied by a significant decrease in serum bile acid levels and the downregulation of bile acid transporters MRP3 and MRP4. These findings, combined with the fact that bile acid metabolism is intricately linked with colitis [7], suggest that matrine’s therapeutic effects are directly tied to its ability to engage this now clinically validated JAK2-bile acid axis.

As for the specific effects of matrine upon colitis, a DSS-induced murine model of colitis was established, and matrine's therapeutic efficacy was distinctly highlighted. The observed improvement in body weight and colon length in matrine-treated mice indicates its potential in mitigating some of the primary physical manifestations of colitis, given that weight loss and reduced colon length are hallmark symptoms of colitis, reflecting the severity of intestinal inflammation and damage [28]. The histopathological analysis further reinforced these findings, where matrine treatment showed notable amelioration in the structural integrity and inflammation of the colon tissues. As mentioned above, matrine could inhibit ROS-mediated NRLP3 inflammasome activation under different contexts [16–18]. In this study, the analysis of inflammatory markers and oxidative stress indicators yielded compelling insights. In both in vivo and in vitro experiments, the reduction in levels of critical pro-inflammatory mediators, including IL-1β, TNF-α, and IL-6 [29, 30], alongside oxidative stress markers including MPO, NO, and MDA [31, 32], post-matrine treatment, underlines its anti-inflammatory and antioxidative properties. Notably, our study also directly confirmed matrine's ability to suppress intracellular ROS, a key aspect of its antioxidant properties given the central role of oxidative stress in colitis progression [33]. This is particularly relevant in the context of colitis, where inflammation and oxidative stress play central roles in disease progression [34]. Thus, matrine’s ability to modulate these factors could be a crucial aspect of its therapeutic action.

Moreover, this study demonstrates that matrine modulates bile acid metabolism in colitis model mice and mouse intestinal epithelial cells. In both the colitis mouse model and LPS-stimulated intestinal epithelial cells, matrine treatment significantly increased the expression of bile acid transport-related factors, including MRP3, MRP4, OSTα, and OSTβ, and bile acid receptor FXR, and restored serum bile acid levels. Bile acids synthesized de novo in the liver undergo amidation to form bile salts. MRP3/ABCC3 and MRP4/ABCC4 provide the sinusoidal efflux function. The heterodimeric organic solute transporter (OSTα/β)/SLC51A/B is a facilitative transporter that may mediate transport into and out of the hepatocyte [35]. A previous study demonstrated that the main ileal bile acid uptake transporter, the apical sodium-dependent bile acid transporter, was downregulated in active CD and UC. In UC, pancolitis was associated with altered expression of major bile acid transporters, including MRP3, MRP4, multidrug resistance gene 1, and OSTα/β, in the descending colon [36]. Meanwhile, matrine was reported to mitigate acute cholestasis and to exert notable hepatoprotective effects in rats [37, 38], suggesting its role in bile acid metabolism. In this study, matrine increases the expression levels of these bile acid metabolism regulators, therefore elevating intestinal tissue bile acid levels. As mentioned above, colitis patients experience bile acid malabsorption and dysbiosis of intestinal flora [7, 39]; therefore, matrine's ability to reverse bile acid dysregulation and intestinal flora dysbiosis might be of considerable therapeutic value. Moreover, emerging evidence indicates that bile acids are not merely metabolic detergents but also potent signaling molecules that interact with receptors such as FXR and TGR5 expressed on intestinal epithelial cells and immune cells, including macrophages and other innate populations [40]. The activation of these bile acid receptors has been shown to modulate immune responses and contribute to the maintenance of intestinal homeostasis. For example, bile acid signaling through FXR can attenuate NF-κB-mediated inflammatory cytokine production and promote epithelial barrier integrity, while TGR5 activation influences macrophage cytokine release and reduces pro-inflammatory responses [41]. In colitis, dysbiosis-linked alterations in the bile acid pool altered receptor activation, potentially amplifying inflammatory signaling and disrupting immune tolerance. For instance, reduced levels of secondary bile acids in dysbiotic colitis microbiota have been shown to affect bile acid receptors such as TGR5 and are associated with increased mucosal inflammation [42]. Based on this body of work, a testable hypothesis arising from our findings is that matrine’s restoration of bile acid homeostasis enhances FXR/TGR5 signaling, which in turn modulates intestinal immune cell activity and limits excessive cytokine production in colitis. Future studies could directly assess FXR/TGR5 signaling activation status and downstream immune signaling in matrine-treated models or determine whether pharmacological manipulation of these receptors synergizes with matrine’s effects.

The central role of the JAK-STAT pathway in colitis, which is critical for mediating cytokine functions [43], was definitively confirmed in our study as the underlying mechanism for matrine’s action. Instead of being a mere possibility, matrine’s therapeutic effects were demonstrated to be JAK2-dependent. IL-6 is a well-established upstream activator of the JAK2/STAT3 cascade [44]. Our experiments showed that JAK2 overexpression alone sufficed to induce inflammation by promoting inflammatory markers (IL-1β, TNF-α, IL-6), oxidative stress, and bile acid dysregulation in intestinal cells. This result supports the concept that increased JAK2 activity can drive autocrine amplification of inflammatory signaling, including enhanced IL-6 production, which in turn reinforces JAK2/STAT3 pathway activation as part of a positive feedback loop in inflammatory contexts. Crucially, the rescue experiment revealed that these pathogenic effects were reversed, and matrine's benefits were restored upon inhibition of this pathway. This work cements the JAK2/STAT3 axis as a key therapeutic pathway and aligns with current therapeutic strategies for colitis, where multiple JAK inhibitors with varying selectivity, such as Upadacitinib, have been investigated for treating UC [45].

While these findings are robust, this study was hindered by limitations, including the use of a DSS model that primarily mimics ulcerative colitis and the focus on epithelial cells. Future research should therefore explore matrine's efficacy in Crohn's disease models, investigate its direct effects on gut-resident immune cells, and examine how restoring bile acid metabolism with matrine could influence the gut microbiome. Although matrine has shown therapeutic effects in preclinical models, it also exhibits dose- and duration-dependent toxicity, with evidence of hepatotoxicity and other adverse effects at higher or prolonged exposures [46], indicating that long-term safety and toxicity profiling will be essential for future clinical translation.

In conclusion, this study provides compelling evidence for matrine's therapeutic potential in colitis, acting through a novel, multifaceted mechanism centered on the JAK2 signaling pathway. For the first time, our study demonstrates that matrine directly engages JAK2 to suppress inflammation and oxidative stress both in vivo and in vitro, with mechanistic confirmation that this effect is JAK2‑dependent, as matrine’s ability to restore bile acid homeostasis and reduce inflammatory mediators was reversed by JAK2 overexpression. The clinical significance of this matrine-related JAK2/STAT3 axis is underscored for the first time by our findings in human UC patients, which confirm that JAK2 is upregulated while bile acid metabolism is dysregulated, mirroring our preclinical results. In addition to inflammation, this work uniquely links restoration of bile acid transporter expression and metabolic regulation with JAK2/STAT3 modulation in colitis, an area that has not been comprehensively addressed. Therefore, matrine represents a promising therapeutic candidate that addresses both the metabolic and inflammatory facets of colitis, moving beyond simple symptom relief to target a clinically validated, fundamental driver of the disease's pathophysiology.

## Data Availability

The data and materials supporting the current study are available from the corresponding author upon reasonable request.

## Abbreviations

AGEs: Advanced glycation end products
CD: Crohn’s disease
CST: Cell Signaling Technology
DSS: Dextran sulfate sodium
DAB: Diaminobenzidine
ECL: Enhanced Chemiluminescence
GO: Gene Ontology
H&E: Hematoxylin and eosin
IBD: Inflammatory bowel disease
IHC staining: Immunohistochemical staining
IgG: Immunoglobulin G
JAK2 oe: JAK2-overexpressing
MDA: Malondialdehyde
MPO: Myeloperoxidase
NO: Nitric oxide
NO2 −: Nitrite
NO3 −: Nitrate
NLRP3: NLR family pyrin domain containing 3
OST: Organic solute transporter
PRRSV: Porcine reproductive and respiratory syndrome virus
RT: Room temperature
T-CHO: Total cholesterol
UC: Ulcerative colitis
